# Nomogram Prediction for the Risk of Diabetic Foot in Patients With Type 2 Diabetes Mellitus

**DOI:** 10.3389/fendo.2022.890057

**Published:** 2022-07-13

**Authors:** Jie Wang, Tong Xue, Haopeng Li, Shuai Guo

**Affiliations:** ^1^ Department of Orthopedic Surgery, Second Affiliated Hospital of Xi’an Jiaotong University, Xi’an, China; ^2^ Department of Neonatology, Second Affiliated Hospital of Xi’an Jiaotong University, Xi’an, China

**Keywords:** type 2 diabetes mellitus (T2DM), diabetic foot, orthopedics, nomogram, individual risk prediction model

## Abstract

**Aims:**

To develop and validate a nomogram prediction model for the risk of diabetic foot in patients with type 2 diabetes mellitus (T2DM) and evaluate its clinical application value.

**Methods:**

We retrospectively collected clinical data from 1,950 patients with T2DM from the Second Affiliated Hospital of Xi’an Jiaotong University between January 2012 and June 2021. The patients were divided into training cohort and validation cohort according to the random number table method at a ratio of 7:3. The independent risk factors for diabetic foot among patients with T2DM were identified by multivariate logistic regression analysis. Then, a nomogram prediction model was developed using the independent risk factors. The model performances were evaluated by the area under the receiver operating characteristic curve (AUC), calibration plot, Hosmer–Lemeshow test, and the decision curve analysis (DCA).

**Results:**

Multivariate logistic regression analysis indicated that age, hemoglobin A1c (HbA1c), low-density lipoprotein (LDL), total cholesterol (TC), smoke, and drink were independent risk factors for diabetic foot among patients with T2DM (*P* < 0.05). The AUCs of training cohort and validation cohort were 0.806 (95% CI: 0.775∼0.837) and 0.857 (95% CI: 0.814∼0.899), respectively, suggesting good discrimination of the model. Calibration curves of training cohort and validation cohort showed a favorable consistency between the predicted probability and the actual probability. In addition, the *P* values of Hosmer–Lemeshow test for training cohort and validation cohort were 0.826 and 0.480, respectively, suggesting a high calibration of the model. When the threshold probability was set as 11.6% in the DCA curve, the clinical net benefits of training cohort and validation cohort were 58% and 65%, respectively, indicating good clinical usefulness of the model.

**Conclusion:**

We developed and validated a user-friendly nomogram prediction model for the risk of diabetic foot in patients with T2DM. Nomograms may help clinicians early screen and identify patients at high risk of diabetic foot.

## Introduction

Type 2 diabetes mellitus (T2DM), previously referred to as noninsulin-dependent diabetes or adult-onset diabetes and accounting for 90%–95% of all diabetes, is a disease caused by a gradual decrease in insulin secretion from β cells in the context of insulin resistance (1, 2). T2DM is a disease involving the interaction between genetic and environmental risk factors leading to the underlying pathophysiology of beta cell dysfunction as well as insulin resistance in liver and muscle (3, 4). Poorly controlled T2DM can lead to chronic diabetic complications such as microangiopathy (retinopathy and kidney disease), atherosclerotic cardiovascular disease, peripheral neuropathy (sensory dysfunction), and diabetic foot (5–10). In the above complications, the diabetic foot negatively affects the quality of both work and life of patients. The decline in daily living activities of patients with diabetic foot results in substantial physical and psychological burdens on the patients.

Diabetic foot is one of the most serious and costly chronic complications of diabetes. It refers to foot ulcer, infection, or deep tissue destruction related to peripheral neuropathy in the lower extremity and peripheral vascular disease (11, 12). Mild diabetic foot patients usually present with foot deformities, hypoesthesia, skin dryness, and loss of skin elasticity. Patients with severe diabetic foot may develop foot ulcers and gangrene. Diabetic foot is the main reason for non-traumatic amputations in orthopedics. At present, there are many studies on the individual risk factors of diabetic foot in patients with T2DM, but no consensus has been reached. Although there are relevant clinical guidelines as the reference basis for the formulation of clinical treatment plans, how to predict the probability of diabetic foot according to risk factors and determine the timing of interventional treatment is an urgent problem to be solved at present.

The nomogram is drawn by the individual risk factors determined by multivariate logistic regression analysis. The nomogram can graphically represent the numerical relationship between specific disease and risk factors and intuitively predict the incidence of adverse events through a scoring system without any complicated calculation formula (13). The nomogram can provide accurate and individualized risk predictions for each individual. It is convenient for clinicians to effectively screen out high-risk patients and timely take interventions. Therefore, this study aimed to develop a nomogram prediction model for the risk of diabetic foot in patients with T2DM. Early screening and identification of high-risk patients can provide the reliable reference basis for early clinical intervention.

## Methods

### Research Subjects

We retrospectively collected and analyzed clinical data from patients with diabetes mellitus from the Second Affiliated Hospital of Xi’an Jiaotong University between January 2012 and June 2021. Baseline-including criteria included (1) T2DM, diagnosis is made according to relevant criteria (fasting plasma glucose ≥ 7.0 mmol/L or 2-h plasma glucose ≥11.1 mmol/L or hemoglobin A1c ≥ 6.5%) (14); (2) both lower extremity arteries (femoral artery, superficial femoral artery, popliteal artery, anterior tibial artery, posterior tibial artery, and dorsalis pedis artery) of patients were examined by color Doppler ultrasonography for intima-media thickness, blood vessel diameter, and filling defects in blood flow; (3) orthopedic examination of the foot and ankle, including visual examination/palpation (skin condition and gait), mobility of foot and ankle (range of motion of the ankle joint, varus/valgus, and pronation/supination), and special examination (ankle anterior drawer test, varus/valgus stress test, and external rotation examination); and (4) patients gave oral informed consent. Baseline-excluding criteria included (1) type 1 diabetes mellitus, (2) gestational diabetes, (3) thromboangiitis obliterans, (4) combined with cancer, and (5) incomplete clinical data.

**Figure 1:**
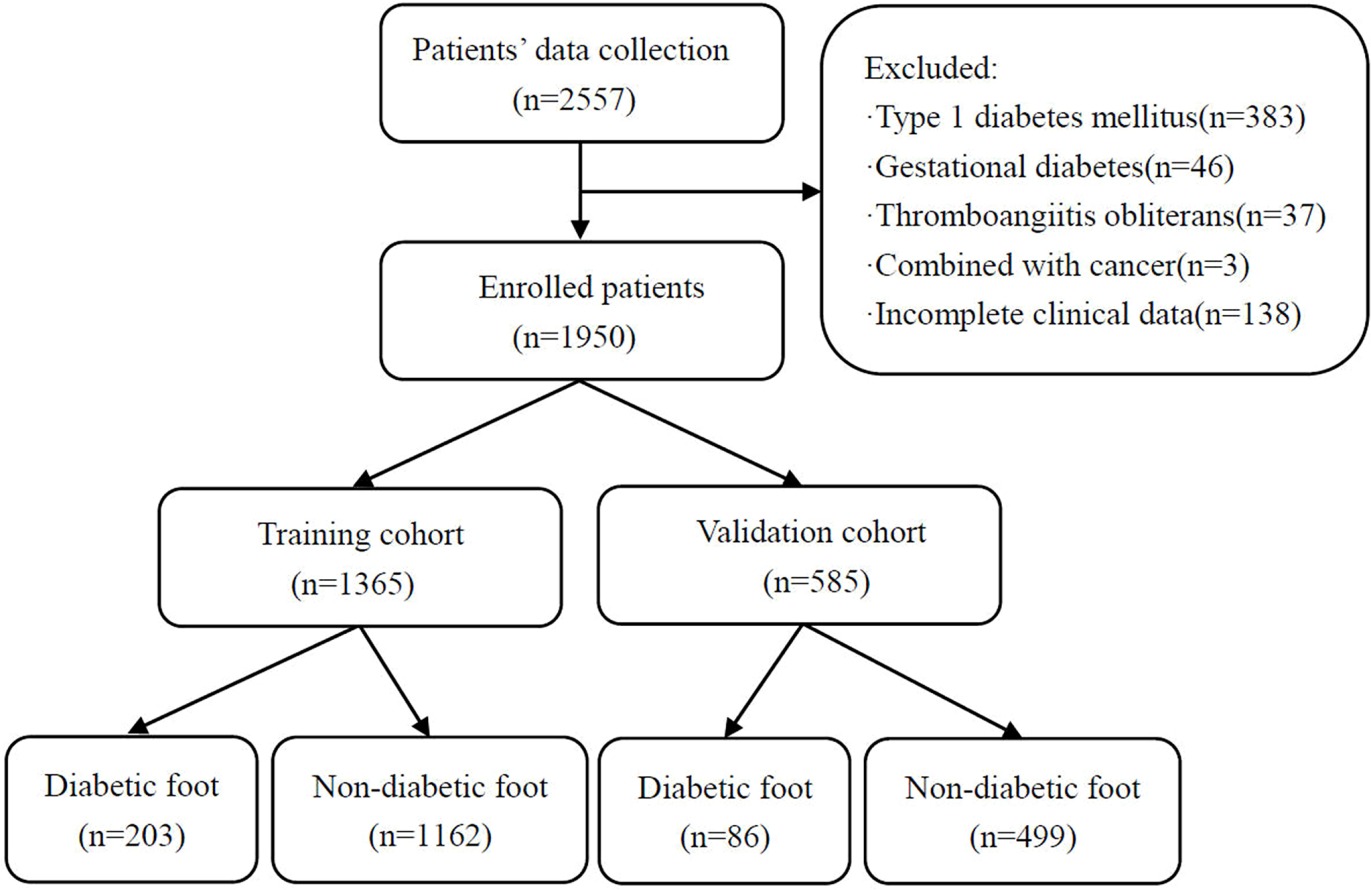
Flowchart of patients included in this study.

After the above screening, a total of 1,950 patients with T2DM were enrolled in the study. The patients were divided into training cohort (*n* = 1,365) and validation cohort (*n* = 585) according to the random number table method at a ratio of 7:3. The detailed flowchart is shown in **Figure 1**.

The study was approved by the medical ethics committee of the Second Affiliated Hospital of Xi’an Jiaotong University (Approval number: 2021234), which was consistent with medical ethics. The study was a retrospective cohort study and the data of included patients were anonymous. Oral informed consent was obtained from each enrolled patient before discharge.

### Observation Indexes

Clinical data including gender, age, course of disease, body mass index (BMI), oral glucose tolerance test (OGTT) 2-h plasma glucose, hemoglobin A1c (HbA1c), low-density lipoprotein (LDL), triglyceride (TG), total cholesterol(TC),smoke, drink, hypertension history, family history of T2DM, and exercise of patients were collected. The above data were collected and checked by three researchers to ensure the completeness and validity of the data.

### Statistical Analysis

Statistical analysis was performed using SPSS software (Version 25.0, USA) and R software (Version 3.6.2, USA). Continuous variables were presented as means ± standard deviation or median (interquartile range). Categorical variables were presented using counts and percentages. Continuous variables were analyzed by *t* test or Mann–Whitney *U* test. Categorical variables were analyzed using the *χ* ^2^ test or Fisher exact test. The independent risk factors for diabetic foot among patients with T2DM were identified by univariate and multivariate logistic regression analysis of the training cohort. Then, a nomogram prediction model was developed using the independent risk factors. The discrimination, calibration, and clinical usefulness of the nomogram prediction model were validated in training cohort and validation cohort. The area under the receiver operating characteristic curve (AUC) and C index were used to evaluate the discrimination. The calibration was evaluated by calibration plot and Hosmer–Lemeshow test. The clinical usefulness was evaluated by cutoff value combined with decision curve analysis (DCA) curve. *P* < 0.05 indicated statistically significant differences.

## Results

### General Characteristics of Research Subjects

A total of 1,950 patients, including 1,365 patients in training cohort and 585 patients in validation cohort, were enrolled in this study (**Table 1**). In the training cohort, 203 patients developed diabetic foot, giving a frequency of 14.9%. In the validation cohort, 86 patients developed diabetic foot, giving a frequency of 14.7%. There were no statistically significant differences in gender, age, course of disease, BMI, etc. between training cohort and validation cohort (*P* > 0.05), indicating comparability between the two groups.

**Table 1:** Characteristics of the patients in the training cohort and validation cohort.

Characteristics	Training cohort (n=1365)	Validation cohort (n=585)	t/Z/χ^2^	*P*
Gender [n(%)]			0.845	0.358
Male	863 (63.2)	357 (61.0)		
Female	502 (36.8)	228 (39.0)		
Age (year)	46.79±2.71	45.12±2.70	0.533	0.601
Course of disease (year)	19.79±1.93	19.10±2.51	1.071	0.298
BMI [n(%)]			2.108	0.349
<18.5kg/m^2^	109 (8.0)	53 (9.1)		
18.5-24 kg/m^2^	846 (62.0)	374 (63.9)		
>24 kg/m^2^	410 (30.0)	158 (27.0)		
OGTT 2h (mmol/L)	14.05±1.85	13.90±1.57	0.441	0.664
HbA1c (%)	10.17±0.96	10.14±0.98	0.679	0.506
LDL (mmol/L)	3.73±1.04	3.79±0.90	1.341	0.180
TG [n(%)]			2.213	0.331
<1.7 mmol/L	478 (35.0)	193 (33.0)		
1.7-2.3 mmol/L	315 (23.1)	153 (26.2)		
>2.3 mmol/L	572 (41.9)	239 (40.8)		
TC (mmol/L)	5.56±1.00	5.58±0.97	0.478	0.633
Smoke [n(%)]			0.244	0.622
No	546 (40.0)	241 (41.2)		
Yes	819 (60.0)	344 (58.8)		
Drink [n(%)]			0.963	0.327
No	642 (47.0)	261 (44.6)		
Yes	723 (53.0)	324 (55.4)		
Hypertension [n(%)]			0.876	0.349
No	802 (58.8)	357 (61.0)		
Yes	563 (41.2)	228 (39.0)		
Family history of type 2 diabetes [n(%)]			0.702	0.402
No	328 (24.0)	151 (25.8)		
Yes	1037 (76.0)	434 (74.2)		
Exercise [n(%)]			1.222	0.269
No	892 (65.3)	367 (62.7)		
Yes	473 (34.7)	218 (37.3)		

BMI, Body Mass Index; OGTT, Oral Glucose Tolerance Test; HbA1c, Hemoglobin A1c; LDL, Low-Density Lipoprotein; TG, Triglyceride; TC, Total Cholesterol.

### Multivariate Logistic Regression Analysis

Univariate logistic regression analysis showed that the risk factors with statistically significant differences were age, course of disease, BMI, HbA1c, LDL, TC, smoke, and drink in training cohort (*P* < 0.05, **Table 2**). Then, the above risk factors were included in the multivariate logistic regression analysis. The results of multivariate logistic regression analysis showed that the independent risk factors for diabetic foot among patients with T2DM were age, HbA1c, LDL, TC, smoke, and drink (*P* < 0.05, **Table 3**).

**Table 2:** Univariate logistic regression analysis of patients in the training cohort.

Characteristics	Diabetic foot group (n=203)	Non-diabetic foot group (n=1162)	t/Z/χ^2^	*P*
Gender [n(%)]			1.001	0.317
Male	122(60.1)	741(63.8)		
Female	81(39.9)	421(36.2)		
Age (year)	47.22±2.98	46.71±2.65	6.081	0.014
Course of disease (year)	20.09±2.00	19.73±1.91	5.848	0.016
BMI [n(%)]			6.426	0.011
<18.5kg/m^2^	15(7.4)	94(8.1)		
18.5-24 kg/m^2^	109(53.7)	737(63.4)		
>24 kg/m^2^	79(38.9)	331(28.5 )		
OGTT 2h (mmol/L)	14.16±1.98	14.03±1.83	0.877	0.349
HbA1c (%)	10.86±0.97	10.05±0.91	112.052	<0.001
LDL (mmol/L)	4.19±0.83	3.65±1.06	42.974	<0.001
TG [n(%)]			2.193	0.139
<1.7 mmol/L	59(29.1)	419(36.0)		
1.7-2.3 mmol/L	54(26.6)	261(22.5)		
>2.3 mmol/L	90(44.3)	482(41.5)		
TC (mmol/L)	5.99±0.75	5.48±1.02	41.716	<0.001
Smoke [n(%)]			6.271	0.012
No	65(32.0)	481(41.4)		
Yes	138(68.0)	681(58.6)		
Drink [n(%)]			7.032	0.008
No	78(38.4)	564(48.5)		
Yes	125(61.6)	598(51.5)		
Hypertension [n(%)]			1.263	0.261
No	112(55.2)	690(59.4)		
Yes	91(44.8)	472(40.6)		
Family history of type 2 diabetes [n(%)]			0.565	0.452
No	53(26.1)	275(23.7)		
Yes	150(73.9)	887(76.3)		
Exercise [n(%)]			1.132	0.287
No	126(62.1)	766(65.9)		
Yes	77(37.9)	396(34.1)		

BMI, Body Mass Index; OGTT, Oral Glucose Tolerance Test; HbA1c, Hemoglobin A1c; LDL, Low-Density Lipoprotein; TG, Triglyceride; TC, Total Cholesterol.

**Table 3:** Multivariate logistic regression analysis of patients in the training cohort.

Variable	B	SE	Wald	OR	95%CI	*P*
Age(year)	0.098	0.031	9.855	1.103	1.038-1.173	0.002
HbA1c (%)	0.920	0.092	100.327	2.509	2.096-3.004	<0.001
LDL (mmol/L)	0.585	0.096	37.078	1.796	1.487-2.168	<0.001
TC(mmol/L)	0.524	0.098	28.877	1.690	1.395-2.046	<0.001
Smoke	0.431	0.179	5.813	1.539	1.084-2.186	0.016
Drink	0.341	0.172	3.931	1.407	1.004-1.971	0.047
Constant	-21.765	2.041	113.677	0.000	–	<0.001

HbA1c, Hemoglobin A1c; LDL, Low-Density Lipoprotein; TC, Total Cholesterol.

### Development of a Diabetic Foot-predicting Nomogram

**Figure 2:**
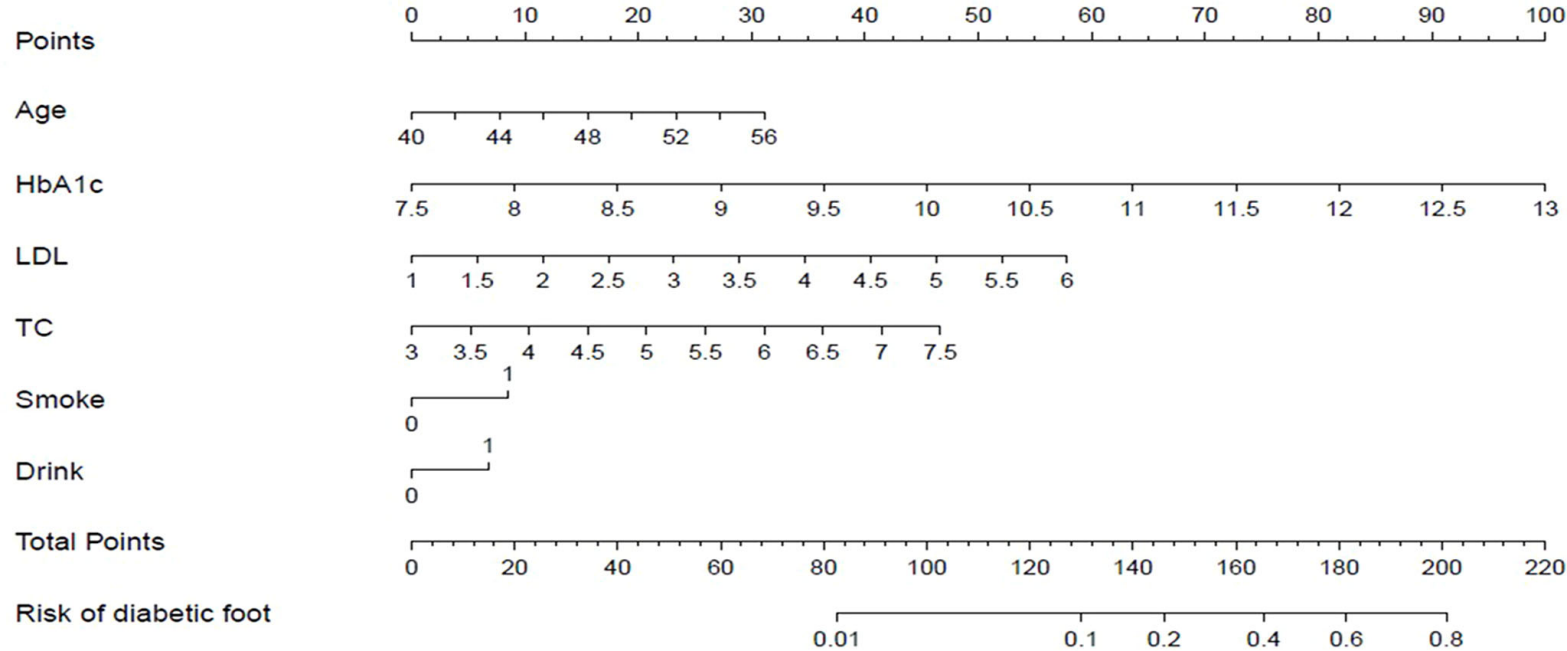
Nomogram prediction for the risk of diabetic foot in patients with T2DM.

A nomogram prediction model for the risk of diabetic foot in patients with T2DM was developed using above independent risk factors (**Figure 2**). The application of the nomogram prediction model was as follows. According to the nomogram, we could obtain the score corresponding to each predictor index, and then the sum of these score was recorded as the total score. The predicted probability corresponding to the total score was the risk of diabetic foot in patients with T2DM.

### Validation of a Diabetic Foot-Predicting Nomogram

#### Discrimination

**Figure 3:**
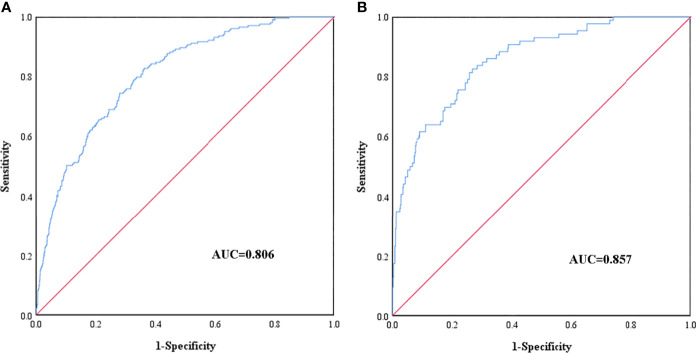
ROC curves of the nomogram prediction for the risk of diabetic foot in patients with T2DM in the training cohort **(A)** and validation cohort **(B)**.

Receiver operating characteristic (ROC) curves of the training cohort and validation cohort were drawn (**Figure 3**). The AUC of the training cohort was 0.806 (95% CI: 0.775∼0.837). The cutoff value was 11.6% (*P* < 0.05). The C index was 0.806. The AUC of the validation cohort was 0.857 (95% CI 0.814∼0.899), (*P* < 0.05). The C index was 0.857. The C indexes of the nomogram prediction model in the training cohort and validation cohort were greater than 0.75, indicating good discrimination of the model.

#### Calibration

**Figure 4:**
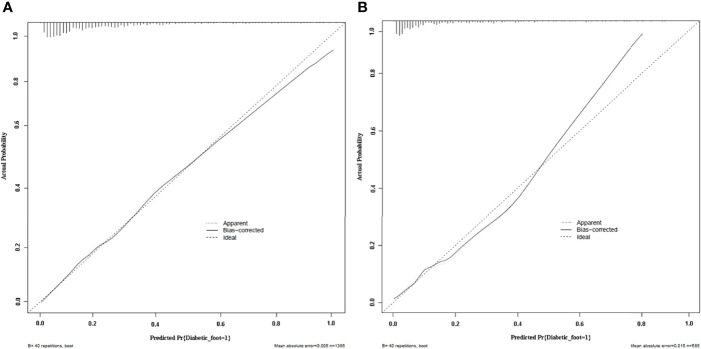
Calibration plots of the nomogram prediction for the risk of diabetic foot in patients with T2DM in the training cohort **(A)** and validation cohort **(B)**.

Calibration curves of the nomogram prediction model in the training cohort and validation cohort showed a favorable consistency between the predicted probability and the actual probability (**Figure 4**). In addition, the results of Hosmer–Lemeshow test of the nomogram prediction model in training cohort and validation cohort were *χ* ^2^ = 4.336 (*P* = 0.826) and *χ* ^2^ = 7.532 (*P* = 0.480), respectively. The *P* values of Hosmer–Lemeshow test of the nomogram prediction model in training cohort and validation cohort were greater than 0.05, suggesting no statistical significance. It indicated that the calibration of the model was high.

#### Clinical usefulness

**Figure 5:**
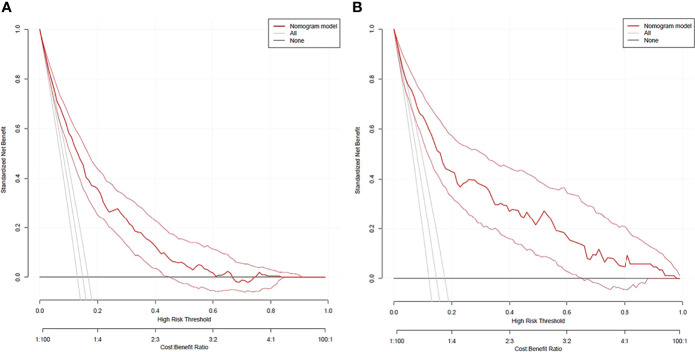
DCA curves of the nomogram prediction for the risk of diabetic foot in patients with T2DM in the training cohort **(A)** and validation cohort **(B)**.

DCA curves of the training cohort and validation cohort were drawn (**Figure 5**). When the threshold probability was in the range of 3%∼62% and 3%∼99%, respectively, the net benefit of patients was higher than that of the other two extreme curves (The horizontal line indicated that no diabetic foot occurred in all patients and no treatment, and the net benefit was 0. The oblique line indicated that all patients developed diabetic foot and received treatment, and the net benefit was a negative slope backslash line.). Within the above range, the nomogram prediction model has good clinical usefulness. The cutoff value (11.6%) obtained from the ROC curve of the training cohort was within the threshold probability range of the above two DCA curves, indicating that the nomogram prediction model has good clinical usefulness. A further analysis of the DCA curves of the nomogram prediction model showed that the net clinical benefit of the training cohort and validation cohort was 58% and 65%, respectively, when 11.6% was set as the threshold probability value for diagnosing diabetic foot and taking intervention. In other words, 58 and 65 of every 100 patients with T2DM who were diagnosed with diabetic foot using the nomogram prediction model in the training cohort and validation cohort would respectively have clinical benefits.

### Visualization Application of a Diabetic Foot-Predicting Nomogram

**Figure 6:**
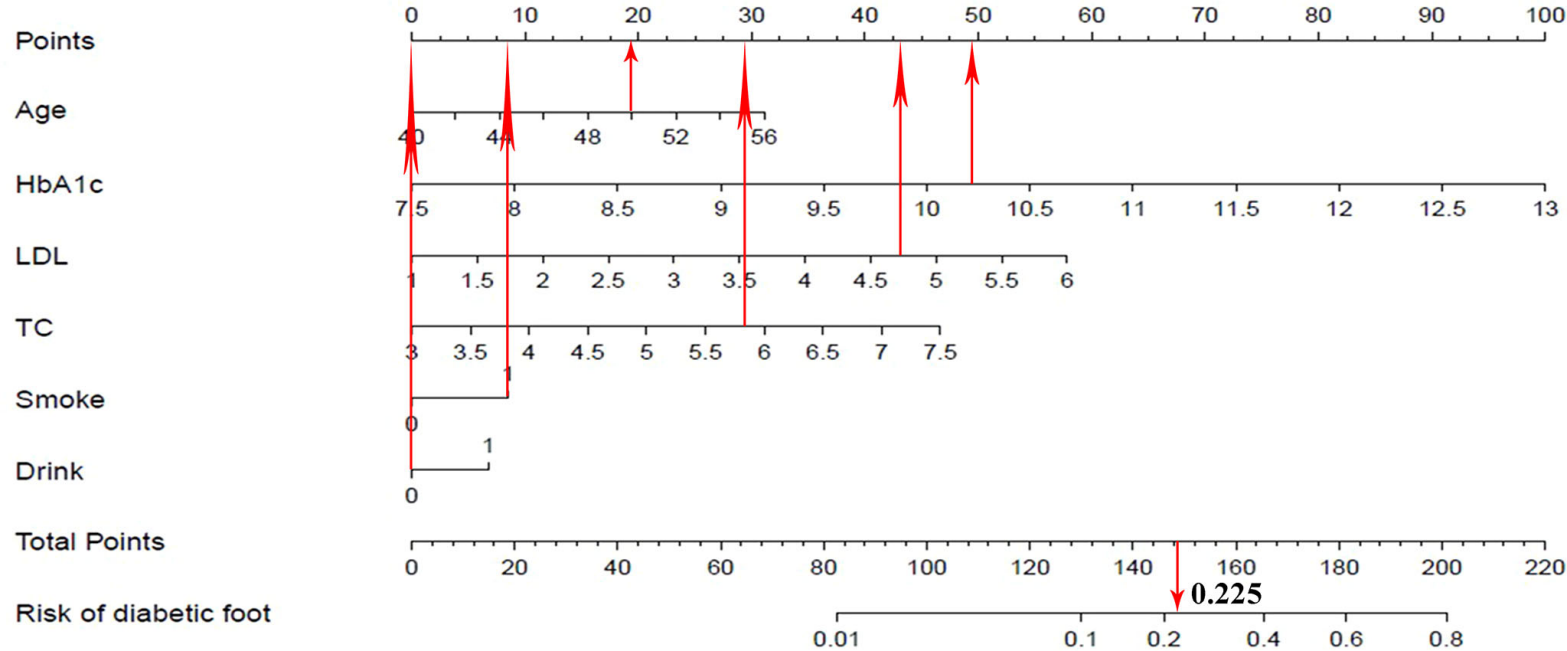
Visualization application of the nomogram prediction for the risk of diabetic foot in patients with T2DM.

Take a patient with T2DM as an example, the relevant clinical data of this patient were as follows: age 50, HbA1c 10.2%, LDL 4.7 mmol/L, TC 5.8 mmol/L, smoke, and drink. According to the nomogram prediction model (**Figure 6**), the predicted risk of diabetic foot for this patient was 22.5%, higher than the threshold probability (11.6%). At this time, according to the DCA curve, we should take intervention to reduce the risk of patients developing diabetic foot.

## Discussion

Diabetic foot was one of the serious complications of diabetes mellitus. Diabetic foot imposed significant economic burdens on patients and society. T2DM accounted for a large proportion of diabetes mellitus. In patients with T2DM, the incidence of diabetic foot was as high as 12% to 15% (15–17). The overall incidence of diabetic foot in patients with T2DM in this study was approximately 14.9%. The incidence was similar to that reported in the above literature. Patients with T2DM who developed diabetic foot often had no obvious clinical symptoms and signs in the early stage, and sometimes only showed their decline of protective sensation. This made it easy to neglect the condition of the patient (18–20). Therefore, how to predict the risk of diabetic foot in patients with T2DM at an early stage and timely take intervention for high-risk patients is crucial.

Most of the current research on the relevant risk factors of diabetic foot has focused on intervening in the progression of diabetic foot to prevent severe ulcers and amputations (21–24). Although these studies are important, we believe that how to prevent diabetic patients from developing diabetic foot is more important and critical. On the one hand, there are few studies in this area. On the other hand, there is no consensus in this regard. In our study, univariate and multivariate logistic regression analysis found that the independent risk factors for diabetic foot in patients with T2DM were age, HbA1c, LDL, TC, smoke, and drink.

The pathogeneses of diabetic foot in patients with T2DM are as follows:

(1) Peripheral neuropathy

Patients with T2DM have various degree of neuropathy of distal lower extremities. Neuropathy of distal lower extremities mainly includes sensory, motor, and autonomic peripheral neuropathy (25). Sensory neuropathy is mainly characterized by the reduction or loss of vibration sensation (pallhypesthesia) and superficial sensation (pressure and touch) as well as subjective paresthesia. The sensation of pain in the foot is substantially declined as a consequence of chronic sensory neuropathy. As a result, the risk of foot trauma is significantly higher (26–29). Foot injuries and ulcers are neglected by patients and doctors due to the lack of pain symptoms (30, 31). Hence, foot injuries and ulcers often go undetected by doctors for weeks. Motor neuropathy of the foot is characterized by muscle atrophy, motor paralysis, and loss of muscle self-reflexes. The combination of sensory and motor peripheral neuropathy results in severe foot load accompanied by gait abnormality. As the disease progresses, the lesions of the foot will worsen due to neuropathy and increased plantar pressure load. Secretion of sweat is dysfunctional by motor paralysis due to autonomic neuropathy. Perspiration dysfunction could lead to dry skin on the foot and a reduced protective skin function, which increases the risk of injury and ulcers. Through multivariate logistic regression analysis, our study found that age, HbA1c, and drink were related to the occurrence of diabetic foot, and their OR values were 1.103, 2.509, and 1.407, respectively. According to our analysis of the patients, the sensory function of distal lower extremities of elderly patients with T2DM was worse than that of young patients, so the risk of foot injuries and ulcers was higher than that of young patients. HbA1c reflects the level of recent plasma glucose control of patients. The increase of HbA1c beyond the normal range usually indicates that the patient’s level of plasma glucose control is not ideal, leading to the occurrence of hyperglycemia. Previous studies showed that metabolic abnormalities due to hyperglycemia cause neuropathy (32). Hyperglycemia leads to nerve damage through four mechanisms, including increased levels of intracellular advanced glycation end products, activation of protein kinase C, hexosamine pathway, and polyol pathway (33). Alcohol has chronic neurotoxic effects (34), especially in patients with T2DM. This has a certain adverse effect on the sensory nerves of distal lower extremities of patients with T2DM, which leads to a decrease in the damage-sensing capacity of foot and increase the risk of diabetic foot in patients with T2DM.

(2) Peripheral vascular disease

Peripheral vascular disease is often present in the course of T2DM. Peripheral vascular disease is an atherosclerotic occlusive disease of the lower extremity. Patients with T2DM have a higher risk of peripheral vascular disease (35). In patients with T2DM, peripheral vascular disease is an important cause of the occurrence and development of diabetic foot (36). Patients with T2DM have a higher incidence of thickened basement membranes of the capillaries, atherosclerosis, endothelial cell hyperplasia, and arteriolosclerosis (37). As a result, patients with T2DM suffer from a lack of blood supply to their arteries. Poor peripheral blood supply can lead to poor wound healing of foot and worsen the condition. Patients with T2DM have reduced blood perfusion in the foot. As a result, the patients are at risk of ulcers and infections and eventually developing diabetic foot. Through multivariate logistic regression analysis, our study found that LDL, TC, and smoke were related to the occurrence of diabetic foot, and their OR values were 1.796, 1.690, and 1.539, respectively. After analyzing the patients, we believed that patients with T2DM would have a great risk of diabetic foot if their LDL and TC were higher than the normal range.LDL, a cholesterol-rich lipoprotein, is one of the risk factors for atherosclerosis (38). After chemical modification, LDL is ingested by phagocytes, forming foamy cells, and remaining in the vascular wall, resulting in a large amount of cholesterol deposition, which contributes to the formation of atheromatous plaque in the arterial wall (39–41). Thus, elevated LDL increases the risk of peripheral vascular disease in patients with T2DM, resulting in poor blood supply to the foot. In this context, diabetic foot ensues. TC is one of the risk factors for atherosclerosis in clinic (42). Therefore, increased TC will increase the risk of peripheral vascular atherosclerosis in patients. Increased TC has adverse effects on the blood supply to the foot of patients with T2DM, leading to the development of diabetic foot. Smoking can reduce the release of prostacyclin in patients, and then platelets tend to adhere to the arterial wall (43, 44). Smoking can also reduce high density lipoprotein cholesterol and increase TC in blood, resulting in atherosclerosis (45–47). Hence, smoking predisposes the patients with T2DM to atherosclerosis, which reduces peripheral blood supply to the foot. This increases the risk of diabetic foot in patients with T2DM.

At present, the preventive measures for diabetic foot mainly include the following. On the premise of glycemic control, the foot of the patients with T2DM should be checked regularly and protected preventatively (such as wearing loose-fitting shoes and socks). In clinical work, how to identify which patients need early clinical intervention is worth pondering. Meanwhile, there is a lack of related research on the nomogram prediction model for the risk of diabetic foot in patients with T2DM. Hence, we developed and validated a nomogram prediction model for the risk of diabetic foot in patients with T2DM and evaluated its clinical application value. In our study, when the cutoff value (11.6%) was taken as the threshold of DCA curve, we observed that the net clinical benefit of patients was higher than that of the other two extreme curves. This suggests that when the risk of diabetic foot is higher than 11.6% predicted by the nomogram prediction model, immediate intervention will benefit the patients clinically. When the risk of diabetic foot is lower than 11.6% predicted by the nomogram prediction model, doctors and patients can temporarily not take intervention and continue to pay attention to the dynamic changes of the disease. This facilitates clinical decision making in patients with T2DM.

There are some limitations to this study. First of all, it is a retrospective study, which requires further prospective studies in the later stage. Secondly, this study is a single-center study at the present stage. If the data of the patients from multiple centers can be included in the later stage to increase the sample size and the range of observed variables, we will further strengthen the nomogram prediction model.

## Conclusion

In conclusion, our study found that the independent risk factors for diabetic foot among patients with T2DM were age, HbA1c, LDL, TC, smoke, and drink. In addition, our study developed an individualized nomogram prediction model, which made the prediction model visualized and easy for clinical application. The nomogram prediction model has good discrimination, calibration, and clinical usefulness in both training cohort and validation cohort. This facilitates early prediction and identification of patients at high risk of developing diabetic foot.

## Data Availability Statement

The raw data supporting the conclusions of this article will be made available by the authors, without undue reservation.

## Ethics Statement

The studies involving human participants were reviewed and approved by The Medical Ethics Committee of the Second Affiliated Hospital of Xi’an Jiaotong University. The patients/participants provided their written informed consent to participate in this study.

## Author Contributions

Study conception and design: JW and HL; data collection and data analysis: JW and TX; manuscript drafting: JW, TX, and SG. All authors were involved in the revision of the manuscript and approved the final version of the paper.

## Funding

This study was supported by the Fundamental Research Funds for the Central Universities (No. ZRZD2017008).

## Conflict of Interest

The authors declare that the research was conducted in the absence of any commercial or financial relationships that could be construed as a potential conflict of interest.

## Publisher’s Note

All claims expressed in this article are solely those of the authors and do not necessarily represent those of their affiliated organizations, or those of the publisher, the editors and the reviewers. Any product that may be evaluated in this article, or claim that may be made by its manufacturer, is not guaranteed or endorsed by the publisher.
